# Post-vaccination SARS-CoV-2 infections and antibody responses after BNT162b2 in patients with severe obesity: a retrospective cohort study

**DOI:** 10.3389/fendo.2026.1759985

**Published:** 2026-03-18

**Authors:** Zehra Kara, Tumay Ak, Ahmet Numan Demir, Rüveyda Akçin, Harika Öykü Dinç, Halit Eren Taşkın, Nesrin Gareayaghi, Bekir Kocazeybek, Volkan Demirhan Yumuk

**Affiliations:** 1 Department of Endocrinology, Metabolism and Diabetes, Cerrahpaşa Medical Faculty, Istanbul University-Cerrahpaşa, Istanbul, Türkiye; 2 European Association for the Study of Obesity–Collaborating Center for Obesity Management, Istanbul, Türkiye; 3 Department of Internal Medicine, Cerrahpaşa Medical Faculty, Istanbul University-Cerrahpaşa, Istanbul, Türkiye; 4 Department of Medical Microbiology, Faculty of Medicine, Istanbul Health and Technology University, Istanbul, Türkiye; 5 Department of Medical Microbiology, Faculty of Medicine, Üsküdar University, Istanbul, Türkiye; 6 Department of General Surgery, Cerrahpaşa Medical Faculty, Istanbul University-Cerrahpaşa, Istanbul, Türkiye; 7 Department of Medical Microbiology, Blood Center Istanbul Sisli Hamidiye Etfal Training and Research Hospital, Istanbul, Türkiye; 8 Department of Medical Microbiology, Cerrahpaşa Medical Faculty, Istanbul University-Cerrahpaşa, Istanbul, Türkiye

**Keywords:** BNT162b2, breakthrough infection, SARS-CoV-2, SARS-CoV-2 IgG, severe obesity

## Abstract

**Aim:**

The aim of this study was to describe the frequency of post-vaccination SARS-CoV-2 infection and to compare SARS-CoV-2 IgG antibody levels between patients with severe obesity and individuals without obesity after two doses of the BNT162b2 vaccine.

**Methods:**

One hundred two consecutive patients with severe obesity seen in the obesity outpatient clinic and 54 individuals without obesity who visited a vaccination outpatient clinic were included in the study. Inactivated Severe Adult Respiratory Syndrome Coronavirus 2 (SARS-CoV-2) IgG levels of these two groups were measured four weeks after administration of two doses of BNT162b2 vaccine. SARS-CoV-2 infection was defined as a PCR-confirmed infection documented in hospital records during the 12-month follow-up after completion of two-dose BNT162b2 vaccination. PCR testing was performed only when participants presented to the hospital with clinical suspicion of COVID-19 and was not conducted as routine screening. Clinical characteristics, protective measures and contact history were also queried.

**Results:**

There was a statistically significant difference in SARS-CoV-2 infection rates after vaccination between the patients with severe obesity (n=28, 27%) and individuals without obesity (n=3, 5%) (p=0.001). In patients with severe obesity, SARS-CoV-2 IgG levels after BNT162b2 vaccination were lower in the group with SARS-CoV-2 infection than in the group without SARS-CoV-2 infection [2697 (1096-8955); 8103 (2208-26903) AUml, respectively] (p=0.008). The antibody levels of those with severe fatigue were lower than those without these complaints [2440 (365-4447); 8955 (2440-16317) AUml, respectively] (p=0.03). A loss of taste or smell was observed in 75% of patients with severe obesity but was not observed in individuals without obesity (p=0.03). In patients with severe obesity, those with taste/smell loss had statistically significantly lower SARS-CoV-2 IgG titres than those without severe obesity [2568 (400-4830); 9526 (2611-16810) AUml, p: 0.02]. Multiple logistic regression analysis revealed a correlation between body mass index (BMI) and having had a SARS-CoV-2 infection after BNT162b2 vaccination (p = 0.028, Exp(B) = 1.072). There was no need for hospitalisation due to SARS-CoV-2 infection and there were no deaths in either group.

**Conclusion:**

Patients with severe obesity had higher rates of SARS-CoV-2 infection after BNT162b2 vaccination compared to individuals without obesity. SARS-CoV-2 IgG levels were lower in patients with severe obesity after BNT162b2 vaccination. Based on these findings, given the higher frequency of post-vaccination infections in the patients with severe obesity, timely booster vaccination policies may be particularly important for this population.

Severe obesity, SARS-CoV-2, BNT162b2, SARS-CoV-2 IgG, breakthrough infection.

**Key points:**

The rate of SARS-CoV-2 infection after vaccination was higher in patients with severe obesity than in individuals without obesity.

It has been found that antibody levels are lower in patients with severe fatigue symptoms than in patients without these symptoms.

Loss of taste or smell was observed at a high rate (75%) in the group with severe obesity, but not in individuals without obesity.

In this study, there were no hospitalisations or deaths after vaccination with BNT162b2 in patients with severe obesity.

Body mass index (BMI) has been found to negatively affect the likelihood of contracting SARS-CoV-2 infection after receiving the BNT162b2 vaccine.

In our study, we emphasise the importance of vaccination in patients with severe obesity.

## Introduction

Severe Acute Respiratory Syndrome Corona Virus 2 (SARS-CoV-2) infection has caused a life-threatening pandemic globally. Since there was no vaccine and treatment at the beginning of the COVID-19 epidemic, humanity faced major health problems (1). The US Food and Drug Administration (FDA) granted emergency use authorization for Pfizer/BioNTech in December 2020. Rapidly developed vaccine received FDA approval in August 2021 in order to reduce cases of SARS-CoV-2 (2).

Individuals with obesity are at higher risk of co-morbidities such as type 2 diabetes (T2DM), hypertension (HT), renal and cardiovascular diseases. In addition, obesity is associated with increased risk of thrombosis, pulmonary dysfunction, and dysfunction of the immune system. Increased ACE2 expression in adipose tissue increases SARS-CoV-2 infection susceptibility and increases disease severity (1, 3).

The results of a meta-analysis showed that risk of SARS-CoV-2 infection was increased in patients with obesity. It was determined that the rates of hospitalization, intensive care unit admission and mechanical ventilation were higher in this patient group. Severe obesity was found to be the highest risk factor for hospitalization in the young population (4). In a meta-analysis of 88 cohort studies, obesity was identified as a negative prognostic factor for community- and hospital-acquired death and/or intensive care unit admission due to SARS-CoV-2 infection (5). In the study by Kalligeros et al., a multivariate regression analysis of patients hospitalised with SARS-CoV-2 infection revealed an association between admission to the intensive care unit and severe obesity (6). Patients requiring mechanical ventilation had a higher prevalence of severe obesity (6).

In this study, we aimed to compare post-vaccination SARS-CoV-2 infections and SARS-CoV-2 IgG antibody responses between patients with severe obesity and individuals without obesity who received two doses of BNT162b2 vaccine.

## Materials and methods

### Patient selection

One hundred and two consecutive patients with severe obesity (BMI≥40kg/m^2^) who visited the Center for Obesity Management (COM) at Istanbul University-Cerrahpaşa, Cerrahpaşa Medical Faculty Hospitals, between August and November 2021 were enrolled. The study included 54 individuals who visited the vaccination clinic during the same period, were not obese, did not have chronic diseases, did not take regular medication; 44 worked in a social setting and 10 worked from home. Those enrolled in the study were grouped in alignment with the obesity classification criteria of World Health Organization (7). The study group with severe obesity (BMI≥ 40 kg/m^2^, n=104) and the individuals without obesity control group (18.5<BMI<30 kg/m^2^, n=54) have already received two doses of BNT162b2 vaccine.

### Data collection

The clinical characteristics of the patients and control subjects such as weight, height, sex, age and presence of T2DM and HT as well as laboratory results such as fasting blood glucose (FBG) and glycosylated haemoglobin A1c (HbA1c) were obtained from the records. Information on the symptoms of patients who experienced SARS-CoV-2 infection after BNT162b2 vaccination, including fever, cough, sore throat, nasal congestion, runny nose, fatigue, extreme weakness, shortness of breath, loss of sense of taste/smell, and symptom duration of 4 days or less/more than 4 days, was obtained from their files. The variable “Four days or less/more than four days” indicates the duration of the acute symptoms (days from the onset of symptoms to significant improvement) (8, 9). In the analysis, this variable was divided into two categories: ≤4 days and >4 days. Data from the literature indicate that the symptom burden/viral dynamics during the acute phase of SARS-CoV-2 infection after vaccination are mainly concentrated in the first 3–5 days and that both infectivity and the acute phase are of short duration (10, 11); therefore, 4 days was used as the threshold.

Peripheral blood samples were obtained 28 days after the last dose of BNT162b2 vaccine. Samples were studied at Medical Microbiology Laboratories, Serology Unit of Cerrahpaşa Medical Faculty. IgG antibody responses specific to Receptor Binding Domain (RBD) region of the virus were quantitatively determined.

All subjects were questioned for SARS-CoV-2 infection one year after two doses of BNT162b2 vaccine and the diagnosis was confirmed by retrospective COVID Polymerase Chain Reaction (PCR) test. Information on the time of infection, how it was acquired, how long it lasted, severity of symptoms and prevention methods applied were recorded. Follow-up was defined as 12 months after completion of the second dose of BNT162b2 vaccination. SARS-CoV-2 infection was defined as a PCR-confirmed infection documented in hospital records during follow-up. PCR testing was performed only when participants presented to the hospital with clinical suspicion of COVID-19 and was not conducted as routine screening. Therefore, participants who did not seek medical care could not be detected as cases in our dataset. Participants were also asked by telephone about the use of at-home testing during follow-up; however, none reported performing home tests.

### Inclusion criteria

Individuals aged 18–90 years who have had two doses of BNT162b2 vaccine 3–4 weeks apart and BMI>18.5.

### Exclusion criteria

Patients with a diagnosis of immunodeficiency disorders, oncological and haematological malignancies, patients receiving corticosteroid, chemotherapy and/or immunotherapy, pregnant women and individuals under 18 years of age were not included in the study.

### SARS-CoV-2 IgG NCP antibody test

The nucleocapsid protein (NCP) facilitates the release of viral ribonucleic acid (RNA) through capsid uncoupling after the virus enters the cell and plays a crucial role in the SARS-CoV-2 life cycle by integrating viral genomic RNA into the ribonucleoprotein complex (12, 13).

Approximately 3 ml of blood taken from volunteers participating in the study into tubes containing vacuum separator gel, was centrifuged at 5000 rpm for 5 minutes, and serum obtained was transferred to microcentrifuge tubes and stored at -20 °C until the study day. On the day of the test, serum samples were first brought to +4 °C, then to room temperature (+18 °C, +25 °C) and made ready for use. The SARS-CoV-2 IgG test (ARCHITECT IgG test, Abbott, USA), which semi-quantitatively detects IgG antibodies against the Nucleocapsid (NCP) protein of SARS-CoV-2, using the chemiluminescent microparticle immunoassay (CMIA) method, was performed. The results obtained from all sera studied were given as index specimen/calibrator (S/C) units (12).

In a previous study conducted in our microbiology laboratory at Cerrahpaşa Medical Faculty in order to determine the diagnostic performance of antibody tests, the mean NCP IgG (2.03 S/Co) in the acute period of COVID-19 was considered as the cut-off index (14).

### SARS-CoV-2 IgG II quant antibody test

The interaction between the SARS-CoV-2 virus’s spike protein receptor binding domain (RBD) and the ACE2 cell surface protein is essential for the virus to infect cells (15). In the study, the SARS-CoV-2 IgG test, which can quantitatively detect immunoglobulin class G (IgG) antibodies, including neutralizing antibodies against the receptor binding region (RBD) of the spike protein S1 subunit of SARS-CoV-2, using the chemiluminescent microparticle immunoassay (CMIA) method (ARCHITECT IgG II Quant test, Abbott, USA). The results obtained from all serums studied were evaluated as Arbitrary Unit/mL (AU/mL). The concentrations obtained in AUmL were multiplied by the correlation coefficient of 0.142 and converted to the “Binding Antibody Unit (BAUmL)” in the WHO’s International Standard (14) on Anti-SARS-CoV-2 immunoglobulin. Accordingly, concentrations of 50 AUmL or 7.1 BAU/mL and above were considered positive. In addition, it was reported that this test was close to 100% compatible with the plaque reduction neutralization test (PRNT), and a concentration of 1050 AU/mL was associated with a 1:80 dilution of PRNT (16).

### Statistical analysis

The statistical analyses were carried out using the Statistical Package for the Social Sciences (SPSS) software (version 21.0). Data were evaluated for normality with the Kolmogorov-Smirnov test. Continuous variables were expressed as mean ± standard deviation (SD) and/or median (interquartile range [IQR]). The Student’s t-test was used when comparing groups with normal data distribution. Medians were compared with the Mann-Whitney U test and the Kruskal-Wallis test. The results were evaluated at the 95% confidence interval. P value < 0.05 was considered statistically significant.

### Sample size

To test the difference between two independent means with a significance level of 5%, a power of 90%, and an effect size of 0.6, we have used G-power programme and found that the required sample size is 98.

## Results

One hundred and two patients with severe obesity (BMI = 41.2 ± 6 kg/m2, age=42.7 ± 10 years) and 54 individuals without obesity (BMI = 25.2 ± 2.8 kg/m2, age=35 ± 9.5 years) who have received 2 doses of BNT162b2 vaccine were enrolled in the study (**Table 1**). After vaccinating with two doses of BNT162b2, it was determined that 28 of the patients with severe obesity (n=102) and three of the individuals without obesity (n=54) had SARS-CoV-2 infection (p=0.001) during the study period.

**Table 1:** Characteristics of patients with severe obesity and the controls.

	Severe obesity n=102 (65%)	Individuals without obesity n=54 (35%)	p
Age (year)	42.7±10	35±9.5	<0.001
Sex (M/FM)	46/8	36/46	<0.001
BMI (kg/m^2^)	41.2±6	25.2±2.8	<0.001
Healthcare workers	0	2 (3%)	0.08
IVI (month)	8.4±3.1	3±2	0.005
SARS-CoV-2 IgG(AUml)^*^	6634(1808-19930)	8103 (2980-16317)	0.3
*Comorbidities*			
DM	40 (39%)	0	<0.001
HT	27 (26%)	0	<0.001
CVD	3 (3%)	0	0.1
CPD	5 (5%)	0	0.07
HbA1c %	7.4±1.7	–	
FPG (mg/dl)	120±50	–	
Pre-vaccine infection rate, n(%)	11 (10%)	18(33%)	<0.001
Post-vaccine infection rate, n(%)	28 (27%)	3(5%)	0.001
Hospitalization rate	0	0	1
Death rate	0	0	1

M/FM, Male/Female; BMI, Body mass index; IVI, Infection-vaccine time interval; SARS-CoV-2 IgG, Severe Acute Respiratory Syndrome.

Coronavirus 2, HT, Hypertension; DM, Diabetes Mellitus; CVD, Cardiovascular disease; CPD, Chronic pulmonary disease; HbA1c, Glycosylated hemoglobin; FPG, Fasting plasma glucose.

*SARS-CoV-2 IgG results are given as geometric mean. *Since the data were not normally distributed, the median (Inter Quantile Range 25%-75%) value was given.

Severe obesity: BMI≥ 40 kg/m2, Non-obese: BMI 18.5-29.9 kg/m2.

p <0.05 suggested statistical significance.

The demographic, clinical and laboratory characteristics of the groups are shown in **Table 1**. The clinical presentation of patients with severe obesity who developed SARS-CoV-2 infection after two doses of BNT162b2 vaccine, and the control group are given in **Table 2**. In addition, contact and protection from SARS-CoV-2 infection status of the same patient group are in **Table 3**.

**Table 2:** Clinical features and acute symptom duration among participants with post-vaccination SARS-CoV-2 infection.

	Severe obesity n=28 (90%)	Individuals without obesity n=3 (10%)	p
Four days or less, n (%)	9 (32%)	2(66%)	0.3
More than four days, n (%)	19 (67%)	1 (33%)	0.3
Fever, n (%)	20(71%)	2 (66%)	0.9
Cough, n (%)	20(71%)	2(66%)	0.9
Sore throat, n (%)	20(71%)	3 (100%)	0.4
Nasal congestion, n (%)	20 (78%)	2 (59%)	0.9
Runny nose, n (%)	17 (60%)	3 (100%)	0.09
Tiredness, n (%)	24 (85%)	3 (100%)	0.7
Severe fatigue , n (%)	12 (42%)	0	0.1
Dyspnea, n (%)	11 (39%)	1 (33%)	0.8
Loss of sense of taste/smell, n (%)	21 (75%)	0	0.03

p <0.05 suggested statistical significance.

**Table 3:** Contact and protective characteristics of the study and control groups with SARS-CoV-2 infection.

	Severe obesity n=28 (90%)		Individuals without obesity n=3 (10%)	p
History of contact				
Household, n(%)		21(75%)	0	0.03
Community, n(%)		1 (3%)	1(33%)	0.4
Workplace, n(%)		0	0	1
Others, n(%)		0	0	1
Unknown, n(%)		6 (21%)	2 (66%)	0.2
History of the measures taken				
Social distance, n(%)	7 (25%)		2 (66%)	0.2
Hand cleaning, n(%)	18 (64%)		2 (66%)	0.9
Mask use, n(%)	16 (57%)		3(100%)	0.2

p <0.05 suggested statistical significance.

The most common comorbidities in patients with severe obesity were T2DM (39%) and HT (26%). The average HbA1c level in these patients was 7.4% (**Table 1**). In the severe obesity group, SARS-CoV-2 IgG levels in patients with T2DM were statistically similar to those in individuals without T2DM [7331 (1808-19930); 6634 (1808-26903) AUml, respectively] (p=0.8). No statistically significant difference in the T2DM diagnosis rate was found between the group of patients who had a SARS-CoV-2 infection after BNT162b2 vaccination and the group without infection (n=9; n=31, respectively) (p=0.7). In the severe obesity group, there was no statistical difference between the SARS-CoV-2 levels of patients with HT and those without HT [8173(1504-25339); 7000(1929-20940) AUml, respectively] (p=0.9). There was no statistically significant difference in the rate of HT diagnosis between the group of patients who had a SARS-CoV-2 infection after BNT162b2 vaccination and the group without infection (n= 5; n= 22, respectively) (p=0.5).

In patients with severe obesity, SARS-CoV-2 IgG levels after BNT162b2 vaccination were lower in the group with SARS-CoV-2 infection than in the group without SARS-CoV-2 infection [2697 (1096-8955); 8103 (2208-26903) AUml, respectively] (p=0.008). Individuals without obesity, there was no statistical difference between SARS-CoV-2 IgG levels in those who had and did not have SARS-CoV-2 infection after BNT162b2 vaccination [9897 (5431-12088); 8103 (2980-16317) AUml, respectively] (p=0.6).

When we compared the antibody levels of males and females in the two groups of patients with severe obesity and individuals without obesity, the antibody levels of female [6634 (2208-19930)] AUml and male [6634 (1480-19930)] AUml patients in the severe obesity group were statistically similar (p=0.6). There was no statistical difference between the antibody levels of female [7331 (2697-29732) AUml] and male [8103 (2980-16317) AUml] patients in the individuals without obesity group (p=0.9). In subgroup analysis by sex, in women with severe obesity (n=66) SARS-CoV-2 IgG levels were found to be lower in those who had SARS-CoV-2 infection compared to those who did not (n=8) (p=0.008). In men with severe obesity (n=36), antibody levels were found to be lower in those who had SARS-CoV-2 infection compared to those who did not (n=46) (p=0.008).

While severe fatigue (42%) and loss of taste/smell (75%) were observed to a high degree in patients with severe obesity, severe fatigue and loss of taste/smell were not observed in individuals without obesity (p=0.1 and p=0.03 respectively). Looking at the symptoms of patients with SARS-CoV-2 infection in patients with severe obesity, the antibody levels of those with severe fatigue symptoms were lower than those without these symptoms [2440 (365-4447); 8955 (2440-16317) AUml, respectively] (p=0.03). Likewise, in patients with severe obesity, the SARS-CoV-2 IgG level was found to be 2568 (400-4830) AUml in cases with loss of sense of taste/smell complaints and 9526 (2611-16810) AUml in those without this complaint (p= 0.02).

It was found that the history of household exposure (living with an infected individual) of SARS-CoV-2 was higher in the severe obesity group than in the control group (p=0.03). When the groups were asked about their environment, a statistically significant difference was found. In the severe obesity group, 27 (26%) of the patients worked in a social setting, while 77 (74%) spent their time at home. In the individuals without obesity group, 44 (81.5%) of patients worked in a social setting, while 10 (18.5%) spent time at home (p<0.001). In the severe obese group, SARS-CoV-2 infection was observed in 25 of 77 patients who stayed at home after receiving the BNT162b2 vaccine, and in 3 of 27 patients who worked in social settings (p = 0.047). In the control group, 3 out of 44 participants working in social settings had a history of SARS-CoV-2 infection after vaccination with BNT162b2, whereas 10 individuals working from home did not (p=0.4).

When comparing the protective measures of people with and without SARS-CoV-2 infection in the severe obesity group, there was a statistical difference in terms of social distancing 7 (25%); 55 (76%) and mask use 16 (57%); 62 (83%) (p<0.001 and p<0.002 respectively), but no difference was found in terms of hand cleaning 18 (64%); 61 (82%) (p=0.3).

### Regression analysis

In multivariate logistic regression analysis, when we regressed the occurrence of SARS-CoV-2 infection after BNT162b2 vaccination on the independent variables age, sex, Type 2 diabetes mellitus, hypertension, and BMI, we found an association between BMI and the occurrence of SARS-CoV-2 infection (p = 0.028, Exp(B) = 1.072).

## Discussion

In the current study, when comparing the severe obesity group and the individuals without obesity group that had received two doses of the BNT162b2 vaccine, the frequency of PCR-confirmed SARS-CoV-2 infection after vaccination was statistically higher in the severe obesity group. Additionally, a correlation has been found between BMI and having had a SARS-CoV-2 infection. However, this study was not designed to estimate vaccine effectiveness/efficacy because an unvaccinated comparator group was not included and exposure risk was not standardised across groups. Moreover, SARS-CoV-2 infections were identified only through hospital records and PCR testing performed when participants presented to the hospital with clinical suspicion; therefore, asymptomatic or mild infections may have been missed.

In the severe obesity group, SARS-CoV-2 IgG levels at day 28 were significantly lower in participants who subsequently developed PCR-confirmed SARS-CoV-2 infection compared with those who did not. Nevertheless, day-28 SARS-CoV-2 IgG levels reflect early humoral response after vaccination, and this single measurement may not represent long-term immune protection. Therefore, the observed differences in antibody levels between participants with and without subsequent infection should be interpreted cautiously and do not imply causality.

In our cohort, patients with severe obesity who developed SARS-CoV-2 infection after BNT162b2 vaccination more frequently reported taste or smell loss compared with individuals without obesity. In addition, lower antibody levels were observed among patients reporting severe fatigue. These symptom-related findings should be considered descriptive observations and may reflect differences in host immune response and symptom perception; however, due to the limited number of detected infections in the non-obese group, these results should be interpreted with caution.

Chronic inflammation due to excessive adipose tissue in patients with severe obesity impairs lymphocyte functions, antibody response and the effect of other immune cells (17). The resulting immune dysfunction increases the risk of SARS-CoV-2 infection, while at the same time reducing the vaccine response (18, 19). In the group with severe obesity, the rate of SARS-CoV-2 infection before vaccination with BNT162b2 was lower than in individuals without obesity. We explained this result by the fact that our patients with severe obesity did not work in the social environment and were at home during the lockdown. In a study of 24 patients with obesity and 32 healthy adults, antibody responses were evaluated after two doses of BNT162b2 vaccination. The antibody responses of patients with obesity were significantly lower (20). In our study, there was no statistically significant difference in SARS-CoV-2 IgG levels in patients with severe obesity after BNT162b2 vaccination compared to individuals without obesity. In the same study, SARS-CoV-2 infection was observed 7 weeks after vaccination in 3 out of 24 patients with obesity, and the disease progressed with mild symptoms in these patients (20). In our study, SARS-CoV-2 infection was seen in 28 of 102 patients with severe obesity, and this rate was significantly higher than in individuals without obesity. In our multiple regression analysis, an increase in BMI was associated with having had a SARS-CoV-2 infection.

A systematic review of 17 studies found that 6 different SARS-CoV-2 vaccines, including the BNT162b2 vaccine, were less effective in patients with obesity and diabetes (21, 22). In another study, the effectiveness of the BNT162b2 vaccine was found to be similar in individuals with obesity and who are individuals without obesity (23). Post-vaccination SARS-CoV-2 infection was observed more frequently in our patients with severe obesity than in individuals without obesity. In the group with severe obesity, the SARS-CoV-2-IgG levels of those who had SARS-CoV-2 infection were significantly lower than those who did not have the infection. No death was observed in either group. Participants survived the infection with mild symptoms, without the need for hospitalization. However, it has been found that patients with severe obesity suffer more frequently from loss of taste or smell than individuals without obesity.

We know that hyperglycaemia negatively affects the functions of immune cells by glycosylating the receptors (24). It impairs the host’s response to SARS-CoV-2 infection, acquisition of immunity, and formation of immunological memory (24). In parallel with this situation, hyperglycaemia fails to provide and long-term protection of the immune response after vaccination (25). Different results have been reported in studies regarding the effect of comorbidities on vaccine immunogenetics (26–28). In this study, patients with severe obesity had comorbidities, especially T2DM, and the blood glucose of our patients with diabetes was not under control. In the severely obese group, when patients with diabetes and without diabetes were compared, pre-vaccine SARS-CoV-2 infection rates were not different. Furthermore, there was no statistical difference between the antibody levels and SARS-CoV-2 infection rates of patients after BNT162b2 vaccination. While there are studies that found the antibody response of diabetic individuals and healthy adults to be similar (24, 25), there are also publications that found the antibody response to be low in diabetic individuals (22, 29). In a study conducted with vaccinated healthcare workers, it was found that SARS-CoV-2 IgG levels were lower in people with HT than in people without HT (30). In their study of approximately 9000 patients, David et al. performed a multivariate regression analysis to assess the independent factors influencing SARS-CoV-2 IgG titre after vaccination: Hypertension was found to negatively affect antibody levels (31). SARS-CoV-2 has a significantly high binding affinity for angiotensin converting enzyme 2 (ACE-2), which reduces the number of particles required to infect a cell. ACE-2/angiotensin receptor blockers are the drugs of first choice for patients with hypertension (32). These drugs increase the expression of the ACE-2 receptor in the tissues (33). People with comorbidities such as hypertension, diabetes and cardiovascular disease are more susceptible to SARS-CoV-2 infection (34) and we see hypertension as one of the most common comorbidities in studies (35). In this study, we found no statistically significant difference in SARS-CoV-2 IgG titres between patients with and without HT. Among patients who developed SARS-CoV-2 infection after BNT162b2 vaccination, the proportion of patients with and without HT was similar. Our sample size was small, we think we will get more accurate results with larger data.

In the study by Bühler and et al., loss of smell/taste was identified as a potential indicator of COVID-19 infection (36). In a study conducted by Gitman and et al. with 18 adolescent patients, they found a negative correlation between loss of sense of smell and SARS-CoV-2 IgG levels (37). In our study, loss of taste/smell and severe fatigue were not observed in individuals without obesity, but only in patients with severe obesity, and patients with these symptoms had lower SARS-CoV-2 IgG levels than patients without these symptoms.

In a study conducted with healthcare workers who were vaccinated with 2 doses of BNT162b2 vaccine, how much the vaccine reduced the risk of infection was evaluated. The efficacy of the vaccine was maximum in the first 2 months and ranged from 72% to 92%. After 6 months, the effectiveness of the vaccine decreased and was found to be between 22% and 69% (11). In the same study, it was observed that the patients did not have SARS-CoV-2 infection for 6 months after the BNT162b2 vaccine (11). In our study, the median time between vaccination and SARS-CoV-2 infection was 8.4 months in patients with severe obesity and 3.2 months in individuals without obesity. We explained this by the fact that patients with severe obesity adhere to infection prevention guidelines and avoid spending time in an infection-prone environment. Aina and colleagues found that SARS-CoV-2 infection occurred mainly among household members and that mask use prevented infection (38). Our study found that in patients with severe obesity who contracted SARS-CoV-2 infection, transmission originated from household members, while social distancing and mask use reduced transmission.

### Limitations

This study has several limitations. First, due to its retrospective observational design and the absence of an unvaccinated comparator group, we could not estimate vaccine efficacy/effectiveness or infer causality. Second, baseline (pre-vaccination) antibody levels were not available, which limits the ability to distinguish vaccine-induced antibody responses from possible prior asymptomatic infection. Third, SARS-CoV-2 infections were identified through hospital record review and PCR testing was performed only when participants presented to the hospital with clinical suspicion; therefore, asymptomatic or mildly symptomatic infections may have been missed. Finally, IgG levels were measured only once at day 28; therefore, antibody levels should not be interpreted as direct predictors of long-term infection risk. Waist circumference measurement values or HOMA-IR results were not included in our study.

## Conclusions

We found that in people with severe obesity, the consequences of SARS-CoV-2 infection after BNT162b2 vaccination were not severe: The median time to SARS-CoV-2 infection after vaccination was 8 months, and there were no deaths or hospitalisations among patients. In our patients with severe obesity, it was found that SARS-CoV-2 IgG levels were lower in patients with SARS-CoV-2 infection after BNT162b2 vaccination than in those without. Given the higher observed post-vaccination infection frequency in the severe obesity group, preventive strategies and timely booster policies may be particularly important in this population. Larger prospective studies with standardised exposure assessment are needed to better define correlates of protection and clinical effectiveness in severe obesity.

## Data Availability

The original contributions presented in the study are included in the article/supplementary material. Further inquiries can be directed to the corresponding author.
