# Correction: Post-vaccination SARS-CoV-2 infections and antibody responses after BNT162b2 in patients with severe obesity: a retrospective cohort study

**DOI:** 10.3389/fendo.2026.1834750

**Published:** 2026-04-15

**Authors:** Zehra Kara, Tumay Ak, Ahmet Numan Demir, Rüveyda Akçin, Harika Öykü Dinç, Halit Eren Taşkın, Nesrin Gareayaghi, Bekir Kocazeybek, Volkan Demirhan Yumuk

**Affiliations:** 1 Department of Endocrinology, Metabolism and Diabetes, Cerrahpaşa Medical Faculty, Istanbul University-Cerrahpaşa, Istanbul, Türkiye; 2 European Association for the Study of Obesity–Collaborating Center for Obesity Management, Istanbul, Türkiye; 3 Department of Internal Medicine, Cerrahpaşa Medical Faculty, Istanbul University-Cerrahpaşa, Istanbul, Türkiye; 4 Department of Medical Microbiology, Faculty of Medicine, Istanbul Health and Technology University, Istanbul, Türkiye; 5 Department of Medical Microbiology, Faculty of Medicine, Üsküdar University, Istanbul, Türkiye; 6 Department of General Surgery, Cerrahpaşa Medical Faculty, Istanbul University-Cerrahpaşa, Istanbul, Türkiye; 7 Department of Medical Microbiology, Blood Center Istanbul Sisli Hamidiye Etfal Training and Research Hospital, Istanbul, Türkiye; 8 Department of Medical Microbiology, Cerrahpaşa Medical Faculty, Istanbul University-Cerrahpaşa, Istanbul, Türkiye

**Keywords:** BNT162b2, breakthrough infection, SARS-CoV-2, SARS-CoV-2 IgG, severe obesity

The affiliation of Bekir Kocazeybek was erroneously published as “Department of Medical Microbiology, Faculty of Medicine, Istanbul Health and Technology University, Istanbul, Türkiye”. The affiliation of Bekir Kocazeybek should be “Department of Medical Microbiology, Cerrahpa̧sa Medical Faculty, Istanbul University-Cerrahpaşa, Istanbul, Türkiye”.

The original version of this article has been updated.

